# A fast approach to detect gene–gene synergy

**DOI:** 10.1038/s41598-017-16748-w

**Published:** 2017-11-27

**Authors:** Pengwei Xing, Yuan Chen, Jun Gao, Lianyang Bai, Zheming Yuan

**Affiliations:** 1 Hunan Engineering & Technology Research Center for Agricultural Big Data Analysis & Decision-making, Hunan Agricultural University, Changsha, Hunan 410128 China; 2 Hunan Provincial Key Laboratory for Biology and Control of Plant Diseases and Insect Pests, Hunan Agricultural University, Changsha, Hunan 410128 China; 3 Department of Biochemistry and Molecular Biology, University of Arkansas for Medical Sciences, Little Rock, 72205 USA; 4 Biotechnology Research Center, Hunan Academy of Agricultural Sciences, Changsha, Hunan 410125 China

## Abstract

Selecting informative genes, including individually discriminant genes and synergic genes, from expression data has been useful for medical diagnosis and prognosis. Detecting synergic genes is more difficult than selecting individually discriminant genes. Several efforts have recently been made to detect gene-gene synergies, such as dendrogram-based *I*(*X*_1_; *X*_2_; Y) (mutual information), doublets (gene pairs) and *MIC*(*X*_1_; *X*_2_; *Y*) based on the maximal information coefficient. It is unclear whether dendrogram-based *I*(*X*_1_; *X*_2_; *Y*) and *doublets* can capture synergies efficiently. Although MIC(*X*_1_; *X*_2_; *Y*) can capture a wide range of interaction, it has a high computational cost triggered by its 3-D search. In this paper, we developed a simple and fast approach based on *abs* conversion type (*i.e*. Z = |*X*_1_ − *X*_2_|) and *t*-test, to detect interactions in simulation and real-world datasets. Our results showed that dendrogram-based *I*(*X*_1_; *X*_2_; *Y*) and *doublets* are helpless for discovering pair-wise gene interactions, our approach can discover typical pair-wise synergic genes efficiently. These synergic genes can reach comparable accuracy to the individually discriminant genes using the same number of genes. Classifier cannot learn well if synergic genes have not been converted properly. Combining individually discriminant and synergic genes can improve the prediction performance.

## Introduction

Selection of informative genes, including individually discriminant genes and synergic genes, from expression data has been useful for medical diagnosis and prognosis. Individual gene ranking techniques such as *t*-test^1^ *etc*. can typically produce a “list of genes” that are correlated with disease^2^. However, they cannot provide insights into the interaction of these genes. According to information theory, the pair-wise interactions *I* (*X*_1_; *X*_2_; *Y*)^3^ is defined as

1 $$I({X}_{1};{X}_{2};Y)=I({X}_{1},{X}_{2};Y)-I({X}_{1};Y)-I({X}_{2};Y)$$

where *I* is the symbol for mutual information, *I* (*X*_1_; *Y*) is the individual effect of gene *X*_1_ relative to phenotype *Y*, *I* (*X*_2_; *Y*) is the individual effect of gene *X*_2_ relative to *Y*, and *I* (*X*_1_, *X*_2_; *Y*) is the joint effect of *X*_1_ and *X*_2_ relative to *Y*. A positive value of *I* (*X*_1_; *X*_2_; *Y*) indicates synergy, while a negative value of *I* (*X*_1_; *X*_2_; *Y*) indicates redundancy.

**Figure 1:**
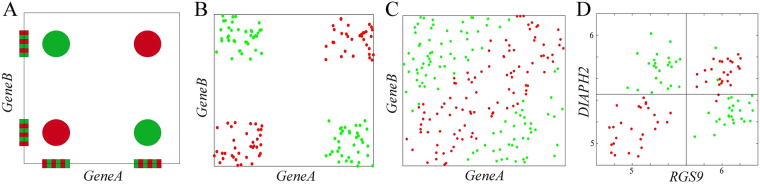
Four typical pair-wise synergies examples. Red and green dots represent cancer and normal samples, respectively.

Figure 1 illustrates four typical pair-wise synergies examples from Watkinson *et al*.^4^ (Fig. 1A,B) and Chen *et al*.^5^ (Fig. 1C,D). Figure 1A–C are generated by simulated data, and Fig. 1D is generated by real-world data. As an example, when the *RSG9* or *DIAPH2* is evaluated individually and separately, neither of these two genes is correlated with cancer. Therefore, genes *RGS9* and *DIAPH2* would not be present in the output of any “individual gene ranking” techniques. However, when the pair-wise interactions is evaluated, the genes *RGS9* -*DIAPH2* are sufficient to distinguish cancer from normal samples (Fig. 1D).

Detecting synergic genes is more difficult than selecting individually discriminant genes. Several efforts have recently been made to detect gene–gene synergies. These efforts often fall into one of the two strategies. One is the non-conversion strategy, which uses formula (1) directly to measure *I*(*X*_1_; *X*_2_; *Y*)^4^ or uses the maximal information coefficient directly to measure MIC(*X*_1_; *X*_2_; *Y*)^5^. The way to discretize continuous variable is the key to estimate the value of mutual information. Binarization, such as the dendrogram-based^4^ technique, simplifies the estimation, and provides simple logical functions in the connection of the genes. However, it may result in information loss and estimation error. Although MIC(*X*_1_; *X*_2_; *Y*)^5^ can capture a wide range of interactions, it has a high computational cost triggered by its 3-D search. The other is the conversion strategy, such as *doublets* ^6^ and top scoring pair (TSP)^7^. They employ a new variable Z derived from the combinations between *X*_1_ and *X*_2_ (*e.g*. for the *sum* type of *doublets*, Z = *X*_1_ + *X*_2_) to measure *I* (*Z*; *Y*) instead of *I*(*X*_1_; *X*_2_; *Y*). This strategy is low computational cost, due to the search space reduced from 3-D to 2-D. However, it is unclear whether this conversion strategy can capture synergies^8^ efficiently.

Inspecting Fig. 1A–C, we found that they share the same pattern and can be characterized by the same function, *Y* = |*X*_1_ − *X*_2_|. The only difference between them is the value ranges of independent variables. Although *Doublets* ^6^ included *sum*, *diff*, *mul* and *sign* conversion types (TSP is similar to *sign*), it, unfortunately, ignored *abs* conversion type.

In this work, we developed a simple and fast approach based on *abs* conversion type and *t*-test, to discover pair-wise synergic genes that are related to cancer. Furthermore, we validated these synergic genes by using classification performance with simulation and real-world datasets. Our results show that these synergic genes can enhance the individually discriminant model and improve the prediction performance. We also demonstrated that these synergic genes should be converted into new variables (Z) prior to be used as input features for classifiers, especially for many pairs of synergistic genes.

## Datasets and Methods

### Datasets

Four binary class datasets are involved in this work. The reference, sample size, number of genes in each dataset, and the number of samples in each class are summarized in Table 1. All gene expression data have been normalized by using the RMA method^9^.

**Table 1:** Four binary class gene expression datasets.

Datasets	Sample size	Number of genes	Reference
Prostate 1	102(52, 50)	12600	Singh, D(2002)^11^
Lung cancer	187 (97, 90)	22,215	Spira, A(2007)^17^; GSE4115
Prostate 2	424 (264, 160)	20,280	Penney, K(2015)^18^; GSE62872
Cardiovascular disease	378 (138, 240)	22,277	Ellsworth, D(2014)^19^; GSE46097

### Conversion types and pair-wise gene rank

Suppose that a dataset has *n* samples and *m* genes, and can be denoted as {*Y*_*i*_, *X*_ij_}, *i* = 1,2,…,*n*; *j* = 1,2,…,*m*. *X*_ij_ represents the expression value of the *j* ^th^ gene (*G*_j_) in the *i* ^th^ sample; and *Y*_i_ represents the class label of *i* ^th^ sample. *Y*_i_ ∈ {0, 1}, 0 denotes cancerous and 1 denotes normal tissue samples. Rank-based methods^7^ are robust to quantization effects and to overcome background differences between gene pairs. Therefore, let *R*_*ij*_ denote the rank of the *i* ^th^ sample in the *j* ^th^ gene, we replace the expression values *X*_ij_ by their ranks *R*_*ij*_ and get a new data matrix {*Y*_*i*_, *R*_ij_}.

For two genes *G*_*p*_ and *G*_*q*_, *Doublets* ^6^ lists four conversion types.

2 $$Sum\,{\rm{c}}{\rm{o}}{\rm{n}}{\rm{v}}{\rm{e}}{\rm{r}}{\rm{s}}{\rm{i}}{\rm{o}}{\rm{n}}\,{\rm{t}}{\rm{y}}{\rm{p}}{\rm{e}}{\rm{:}}{Z}_{is}={R}_{ip}+{R}_{iq}$$

3 $$Diff\,{\rm{c}}{\rm{o}}{\rm{n}}{\rm{v}}{\rm{e}}{\rm{r}}{\rm{s}}{\rm{i}}{\rm{o}}{\rm{n}}\,{\rm{t}}{\rm{y}}{\rm{p}}{\rm{e}}{\rm{:}}{Z}_{is}={R}_{ip}\,-\,{R}_{iq}$$

4 $$Mul\,{\rm{c}}{\rm{o}}{\rm{n}}{\rm{v}}{\rm{e}}{\rm{r}}{\rm{s}}{\rm{i}}{\rm{o}}{\rm{n}}\,{\rm{t}}{\rm{y}}{\rm{p}}{\rm{e}}{\rm{:}}{Z}_{is}={R}_{ip}\times {R}_{iq}$$

5 $$Sign\,{\rm{c}}{\rm{o}}{\rm{n}}{\rm{v}}{\rm{e}}{\rm{r}}{\rm{s}}{\rm{i}}{\rm{o}}{\rm{n}}\,{\rm{t}}{\rm{y}}{\rm{p}}{\rm{e}}{\rm{:}}{Z}_{is}=\{\begin{array}{c}1\,,\,{\rm{if}}\,{R}_{ip}\ge {R}_{iq}\\ 0\,,\,{\rm{if}}\,{R}_{ip} < {R}_{iq}\end{array}$$

We add a new conversion type:

6 $$Abs\,{\rm{c}}{\rm{o}}{\rm{n}}{\rm{v}}{\rm{e}}{\rm{r}}{\rm{s}}{\rm{i}}{\rm{o}}{\rm{n}}\,{\rm{t}}{\rm{y}}{\rm{p}}{\rm{e}}{\rm{:}}{Z}_{is}=|{R}_{ip}\mbox{--}{R}_{iq}|$$

Here, *i* = 1,2,…,*n*; *p* = 1,2,…, *m*; *q* = 1,2,…, *m*; *p* ≠ *q*; *s* = 1,2,…, *m*(*m*−1)/2. Again, we get a new data matrix {*Y*_*i*_, *Z*_*is*_}. For each converted feature *Z*_*s*_, we use the *t*-score, instead of *I* (*Z*; *Y*), to rank the association between Z and *Y*, since *Y* ∈ {0, 1}.

The individually discriminant genes are also ranked by *t*- score.

### Support Vector Machine Classifier and performance evaluation

Each gene pairs and each individually discriminant genes are ranked by *t*- score based on all samples. The Top *N* gene pairs and/or the Top *N* individually discriminant genes are selected as input features. Support Vector Machine (SVM) Classifier is available at http://www.csie.ntu.edu.tw/~cjlin/libsvm/ ^10^. We simply use the average accuracy of five-fold cross-validation (CV) to evaluate the classifier performance as the datasets involved in this paper have balanced numbers of positive and negative samples.

7 $$Accuracy=\frac{{\rm{TP}}+{\rm{TN}}}{{\rm{TP}}+{\rm{FP}}+{\rm{TN}}+{\rm{FN}}}\times 100 \% $$

Here TP, TN, FP, FN denote true positives, true negatives, false positives and false negatives respectively.

## Results and Discussion

### Comparing gene pairs selected by different methods

**Figure 2:**
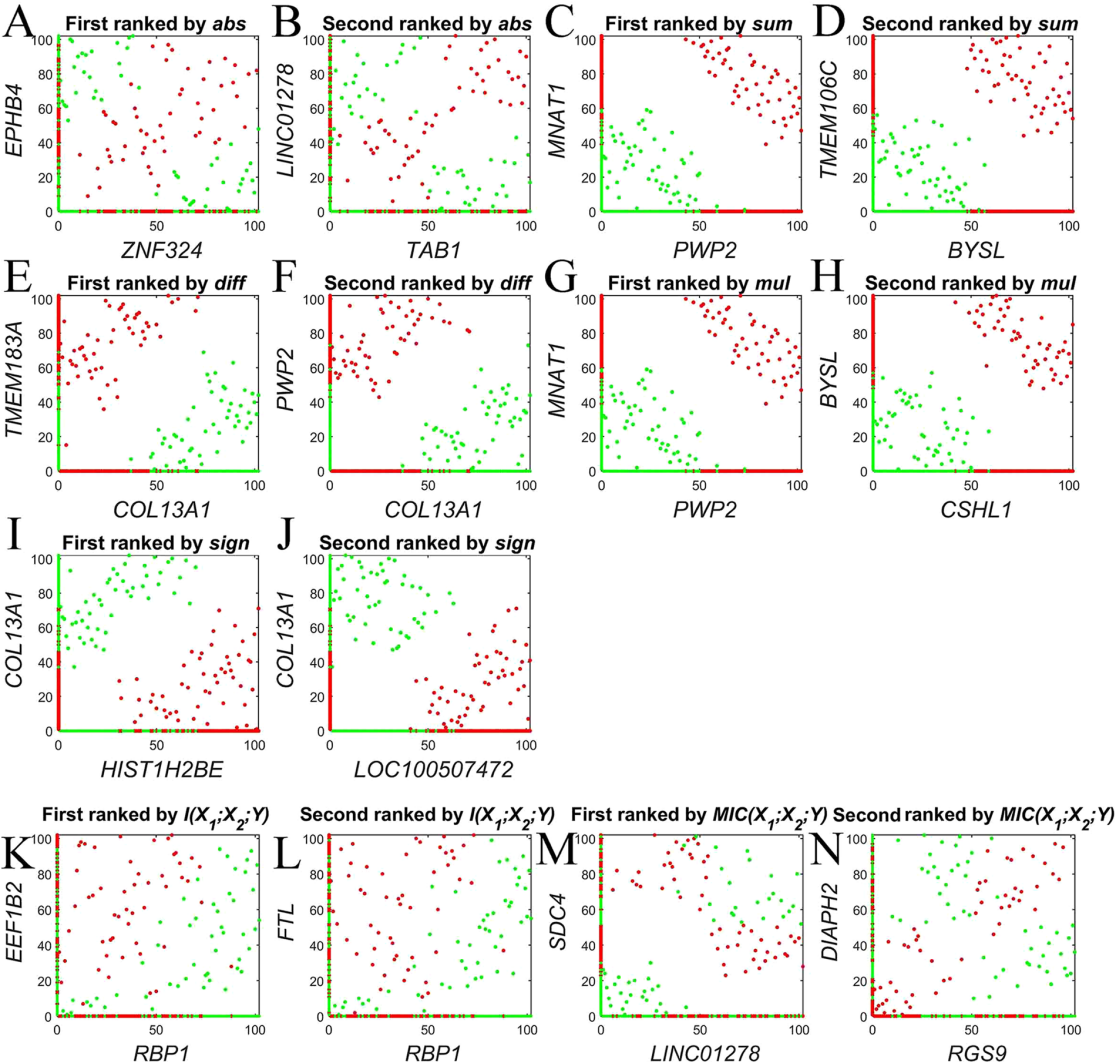
Top2 gene pairs selected by different methods in Prostate1 dataset. Red and green dots represent cancer and control, respectively. Gene expression levels are represented by the ranked values. K and L are from dendrogram-based I(*X*_1_; *X*_2_; *Y*)^4^, M and N are from MIC(*X*_1_; *X*_2_; *Y*)^5^.

Figure 2 illustrates the scatterplot of the top-two gene pairs selected by *abs* conversion type and six reference methods in Prostate1 dataset^11^. In Fig. 2A,B,M and N, although the top-two synergic genes selected by *abs* conversion type and MIC(*X*_1_; *X*_2_; *Y*) are different, they share the same pattern: each individual gene is unrelated to cancer by individual gene evaluation, but the pair-wise genes are sufficient to distinguish the cancer from normal samples. Figure 2C–L are the top-two gene pairs selected from *sum*, *diff*, *mul*, *sign* and dendrogram-based I(*X*_1_; *X2*; *Y*) methods. As an example (Fig. 2C), the higher the gene *PWP2* expression level, the more likely to suffer cancer. The gene *MNAT1* showed similar pattern as PWP2. Thus, these two genes (PWP2 and MNAT1) are related with cancer directly. However, they are individually discriminant rather than synergic genes. In a word, only *abs* conversion type and MIC(*X*_1_; *X*_2_; *Y*) can capture typical pair-wise synergies, dendrogram-based *I*(*X*_1_; *X*_2_; *Y*) and *doublets* are helpless for discovering pair-wise gene interactions.

We then compared the overlaps among the informative genes selected by *Ind*, *Sum*, *Diff*, *Mul*, *Sign* and *Abs* methods (Table 2). Clearly, a considerable number of similar informative genes can be detected by the first five methods. On the contrary, the informative genes selected by *Abs* method have little overlap with the informative genes selected by the others.

**Table 2:** Overlaps among the informative genes selected by different methods in the Prostate1 dataset.

	*Ind*(100)	*Sum*(98)	*Diff*(94)	*Mul*(70)	*Sign*(128)	*Abs*(132)
*Ind*(100)						
*Sum*(98)	35					
*Diff*(94)	36	41				
*Mul*(70)	23	20	21			
*Sign*(128)	25	28	30	18		
*Abs*(132)	1	0	0	0	0	

*Ind*(100): The Top 100 individually discriminant genes selected by *t*-test. *Sum* (98): The Top 100 gene pairs selected by *Sum* conversion type and *t*-test, 98 genes reserved after removing repeated genes; the others as well.

**Figure 3:**
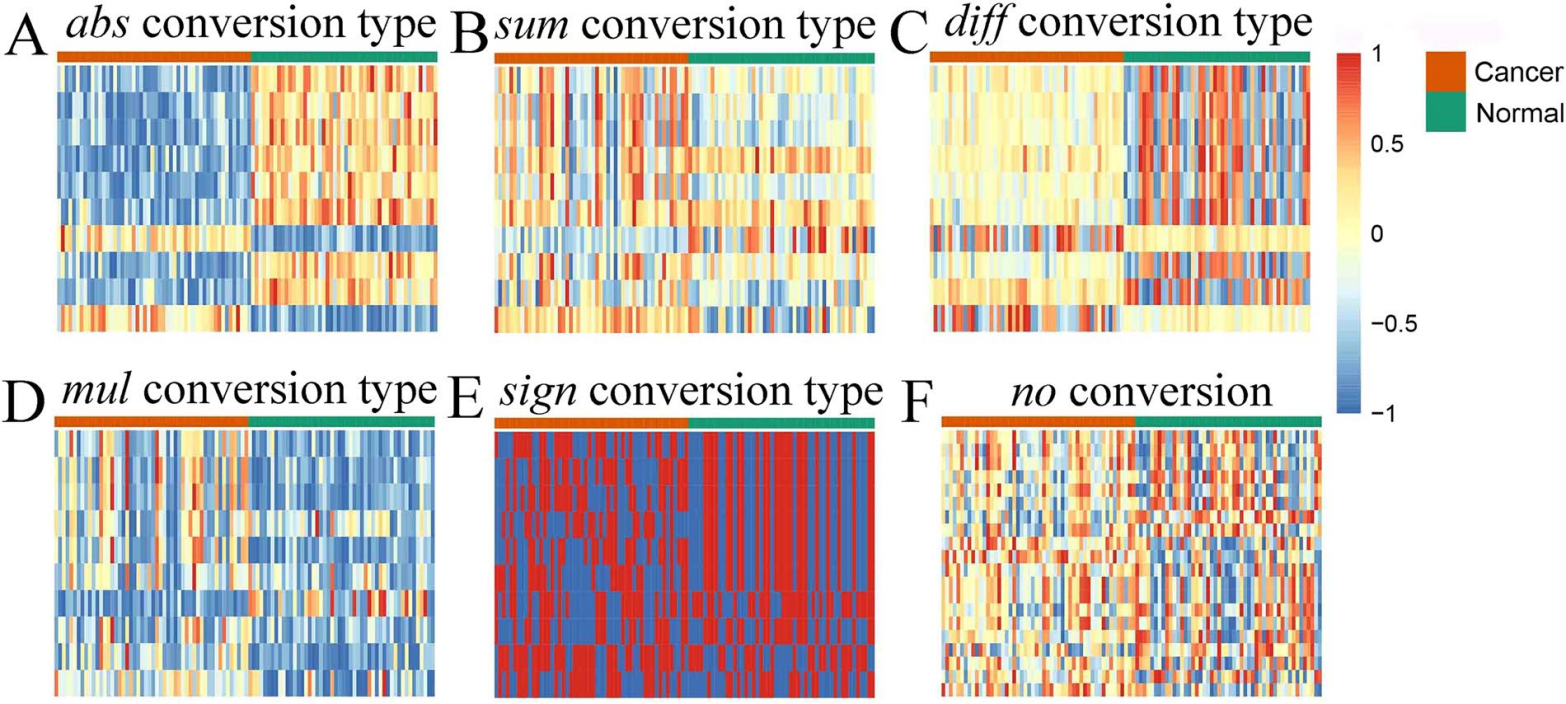
The heat maps generated by the same top10 synergic genes which were selected by *abs* conversion type. Each row corresponds to a pair of genes (**A**–**E**) or a gene (**F**), and each column corresponds to a sample. Gene expression levels are represented by the ranked values, and normalized to [−1, 1].

Given the top10 pair-wise synergic genes (16 genes) selected by *abs* conversion type, Fig. 3 contains the heat maps generated by these genes with different conversion type. Only the heat maps with *abs* conversion type (Fig. 3A) and *diff* conversion type (Fig. 3C) can distinguish cancer from normal samples. In *diff* conversion type, the *Z* values are medium in cancer samples, but they are either low or high in normal samples, and *vice versa*. Therefore, the pair-wise synergic genes converted by *diff* will receive low *t*-scores and cannot be highlighted.

To answer whether the synergic genes selected by *abs* conversion type have any biological relevance to cancer, we further validated the top10 gene pairs (16 genes) according to UniHI^12^ database (http://www.unihi.org/) and PubMed (Table 3). UniHI is an enhanced database for retrieval and interactive analysis of human molecular interaction networks. In Top10 gene pairs, so far we have found two gene pairs (*PARP*1-*HMGB*1 and *CCHCR*1-*GRAP*) that are associated with interaction in UniHI. The interaction between *PARP*1 and *HMGB*1 has been verified by Dara *et al*. (2007)^13^, the activation of *PARP1* induces release of the pro-inflammatory mediator *HMGB1* from the nucleus^13–15^. Of the 16 genes, 15 of them have been reported to relate to cancer. Four of them have been reported to relate to prostate cancer directly. Although *LINC01278* has not yet been reported to relate to cancer, *abs* conversion type suggests that it is an important informative gene. *LINC01278* occurred three times in the top 10 gene pairs (Table 3), and should be given proper attention.

**Table 3:** The top10 synergic genes selected by *abs* conversion type in Prostate1 dataset.

Pair-wise synergic Genes	Related carcinoma and Ref.
*ZNF324–EPHB4*	Breast cancer^20^ *–* Prostate cancer^21^
*TAB1–LINC01278*	Breast cancer^22^ *–* Unreported
*CDH22–LINC01278*	Colorectal cancer^23^ *–* Unreported
*KLF7–EXT1*	Oral carcinoma^24^ *–* Cartilage-capped tumor^25^
*SIPA1L3–LINC01278*	Breast cancer^26^ *–* Unreported
*KLF7–DDR2*	Oral carcinoma^24^ *–* Lung cancer^27^
*MMP23A–DIP2C*	Bladder cancer^28^ – Breast and lung cancer^29^
*CARM1–EPHB4*	Prostate cancers^30^ *–* Prostate cancer^21^
*CCHCR1–GRAP*	Skin cancer^31^ *–* Medullary thyroid carcinoma^32^
*PARP1–HMGB1*	Prostate cancer^33^ *–* Prostate cancer^13^

### Classifier cannot learn well if synergic genes have not been converted properly

Although we get the pair-wise synergic genes based on *abs* conversion type, Fig. 3F suggests that the no conversion feature (*X* or *R*) cannot distinguish cancer from normal samples. It also indicates that the input features for classifiers should be conversion feature *Z* (Fig. 3A). Therefore, we conducted an experiment to further validate this hypothesis. Ten simulation datasets were generated according to Table 4; their prediction accuracy of 5 fold cross-validation is listed in Table 5.

**Table 4:** Ten simulation datasets and their input features.

Dataset	Function	No converted input features	Converted input features
1	*Y* = *|X*_1_ − *X*_2_| = *Z*_1_	{*X*_1_, *X*_2_}	{*Z*_1_}
2	*Y* = *|X*_1_ − *X*_2_| + *|X*_3_ − *X*_4_| = *Z*_1_ + *Z*_2_	{*X*_1_, *X*_2_, *X*_3_, *X*_4_}	{*Z*_1_, *Z*_2_}
…	…	…	…
10	*Y* = *|X*_1_ − *X*_2_| + *|X*_3_ − *X*_4_| + … + *|X*_19_ − *X*_20_| = *Z*_1_ + *Z*_2_ + … + *Z*_10_	{*X*_1_, *X*_2_, *X*_3_, *X*_4_,…, *X*_19_, *X*_20_}	{*Z*_1_, *Z*_2_,…, *Z*_10_}

Here, *X* is assigned with random values between 0 and 1, and *Y* is binarized with the median. Sample size for each dataset is 200.

**Table 5:** Prediction accuracy with converted and not converted input features.

Dataset	SVM-RBF^a^		SVM-linear^b^		SVM-poly^c^		SVM-sig^d^		RF		ANNs		DT	
	*Con*.	*No con*.	*Con*.	*No con*.	*Con*.	*No con*.	*Con*.	*No con*.	*Con*.	*No con*.	*Con*.	*No con*.	*Con*.	*No con*.
1	0.985	0.985	0.990	0.605	1.00	0.56	0.990	0.540	1.00	0.865	1.00	0.975	0.995	0.895
2	0.970	0.905	0.975	0.600	0.985	0.640	0.995	0.455	0.960	0.795	0.990	0.930	0.965	0.785
3	0.985	0.860	0.975	0.465	0.980	0.575	0.975	0.500	0.860	0.780	0.995	0.910	0.900	0.705
4	0.960	0.810	0.925	0.515	0.985	0.400	0.980	0.420	0.850	0.655	0.985	0.825	0.865	0.695
5	0.970	0.790	0.910	0.535	0.965	0.550	0.980	0.460	0.810	0.615	0.995	0.780	0.840	0.600
6	0.945	0.815	0.860	0.500	0.985	0.475	0980	0.485	0.770	0.620	0.990	0.770	0.795	0.615
7	0.940	0.715	0.905	0.530	0.980	0.500	0.980	0.535	0.865	0.610	0.985	0.670	0.795	0.585
8	0.970	0.675	0.955	0.410	0.970	0.455	0.955	0.455	0.760	0.545	0.995	0.695	0.760	0.610
9	0.955	0.660	0.885	0.515	0.960	0.460	0.955	0.435	0.790	0.510	0.990	0.665	0.770	0.580
10	0.955	0.655	0.860	0.480	0.955	0.525	0.975	0.525	0.735	0.520	0.960	0.600	0.750	0.625

Here, *a*: SVM with radial basis function (RBF) kernel; *b*: SVM with linear kernel; *c*: SVM with polynomial kernel; *d*: SVM with sigmoid kernel. RF: Random Forest; ANNs: artificial neuron network; DT: Decision Tree; *Con*: the converted input features; *No con*: the not converted input features.

For the less input features (*e.g* dataset1 and dataset2) (Table 5), all of the seven models perform well by applying with the converted features, whereas only two models (SVM-RBF and ANNs) perform well by applying with the not- converted features. For the larger input features (*e.g* dataset9 and dataset10) (Table 5), although four models (SVM-RBF, SVM-poly, SVM-sig and ANNs) still perform well by applying with the converted features, none of these seven models perform well by applying with the not converted features. Thus, we can conclude that pair-wise synergic genes should be converted into new variables (*Z*) prior to be used as input features for classifiers, especially for many pairs of synergistic genes.

This is a surprising and important discovery. Suppose phenotype *Y* is determined by individually discriminant genes *X*_1_ and *X*_2_, and pair-wise synergic genes *X*_3_–*X*_4_ and *X*_5_–*X*_6_. In other words, the true genetic model is $Y=X1+X2+|X3-X4|+|X5-X6|$, and the true optimal subset is {*X*_1_, *X*_2_, *X*_3_, *X*_4_, *X*_5_, *X*_6_}, *X*_7_–*X*_1000_ are genes unrelated to *Y*. Now we get the dataset {*Y*, *X*_1_, *X*_2_,…, *X*_1000_} and want to construct a genomic prediction model^16^ based on machine learning, but don’t know the true genetic model. Even the individual discriminant genes *X*_1_ and *X*_2_ can be highlighted by *t*-test, and the synergic genes *X*_3_, *X*_4_, *X*_5_ and *X*_6_ can be highlighted by *Abs* conversion type or MIC(*X*_1_; *X*_2_; *Y*), classifier cannot learn well when the input features space is {*X*_1_, *X*_2_, *X*_3_, *X*_4_, *X*_5_, *X*_6_}. It means that learning machine can never tell us the true optimal subset, if synergic genes have not been converted properly. This indicates the complexity of genomic prediction, also provides a new explain for “missing heritability” in GWAS study.

### Combining individually discriminant and synergic genes can improve prediction performance

To further validate the reliability of synergic genes selected by *abs* conversion type, we also evaluated the prediction performance of individually discriminant and synergic genes with three more recent and larger publicly available datasets (Lung, Prostate2 and Cardiovascular) (see Table 1). Meantime, the label randomization tests were performed. The top individually discriminant genes are selected by *t*-test, the top synergic genes are selected by *abs* conversion type + *t*-test. Here, we take the individually discriminant genes and/or converted synergic genes as the input features for the SVM-RBF classifier.

Table 6 illustrates the prediction of accuracy in different schemes of input features. The results show that: 1) By using the individually discriminant genes as input features alone, the average accuracies for Top10_*Ind*, Top20_*Ind* and Top40_*Ind* are 77.30%, 78.74% and 80.36%, respectively. By using the synergic genes as input features alone, the average accuracies for Top5_*Syn*, Top10_*Syn* and Top20_*Syn* are 75.58%, 81.67% and 84.63%, respectively. These indicate that the synergic genes receive comparable accuracy to the individually discriminant genes using the same number of genes. 2) When the input features involves 20 genes, the average accuracies for Top20_*Ind*, Top10_*Syn* and Top10_*Ind* + Top5_*Syn* are 78.74%, 81.67%, and 83.74%, respectively. When the input features involves 40 genes, the average accuracies for Top40_*Ind*, Top20_*Syn* and Top20_*Ind* + Top10_*Syn* are 80.36%, 84.63%, and 85.75%, respectively. These indicate that combining individually discriminant and synergic genes, rather than only using the individually discriminant genes or the synergic genes, can receive better prediction accuracies. 3) The classification performances of the label randomization tests drop to random, it validate the reliability of synergic genes selected by *abs* conversion type.

**Table 6:** Prediction accuracies of 5-fold CV in different schemes of input features (%).

Input features	Lung	Prostate2	Cardiovascular	Average
Top10_*Ind*	74.41 (43.81)	84.20 (64.39)	73.29 (63.22)	77.30 (57.14)
Top20_*Ind*	76.49 (43.31)	85.13 (61.08)	74.59 (61.65)	78.74 (55.35)
Top40_*Ind*	75.93 (46.02)	84.20 (61.09)	80.96 (62.95)	80.36 (56.69)
Top5_*Syn*	76.54 (47.03)	74.52 (62.25)	75.67 (62.99)	75.58 (57.42)
Top10_*Syn*	84.44 (50.28)	76.18 (55.90)	84.40 (61.38)	81.67 (55.85)
Top20_*Syn*	83.98 (47.06)	80.20 (62.96)	89.70 (62.17)	84.63 (57.40)
Top10_*Ind* + Top5_*Syn*	82.33 (48.17)	86.34 (62.27)	82.55 (63.22)	83.74 (57.89)
Top20_*Ind* + Top10_*Syn*	83.91 (40.11)	86.31 (57.54)	87.04 (62.44)	85.75 (53.36)

*Ind* represents the individually discriminant genes, *Syn* represents the synergic genes. A number in parentheses indicates the result of label randomization test.

The minimum number of individually discriminant and synergic genes required in the optimal subset remains to be determined by the further research.

We also compared the prediction performance of the 5 conversion types (Table 7). The results show that the genes selected by *Abs* conversion type have more powerful ability to improve prediction performance for the individually discriminant model than the genes selected by the other conversion types.

**Table 7:** Prediction accuracies of 5-fold CV in different conversion types (%).

Features	Lung	Prostate2	Cardiovascular	Average
Top20_*Ind*	76.49	85.13	74.59	78.73
Top10_*Sum*	80.68	81.61	78.83	80.37
Top10_*Diff*	83.37	85.84	76.97	82.06
Top10_*Mul*	80.81	81.61	79.09	80.50
Top10_*Sign*	78.08	84.68	79.38	80.71
Top10_*Abs*	84.44	76.18	84.40	81.67
Top10_*Sum* + Top20_*Ind*	79.70	85.14	80.42	81.75
Top10_*Diff* + Top20_*Ind*	82.33	84.44	83.33	83.37
Top10_*Mul* + Top20_*Ind*	78.11	**86.55**	79.64	81.43
Top10_*Sign* + Top20_*Ind*	81.35	84.43	76.21	80.66
Top10_*Abs* + Top20_*Ind*	**83.91**	86.31	**87.04**	**85.75**

Top20_*Ind*: The Top20 individually discriminant genes selected by *t*-test. Top10_*Sum*: the Top10 gene pairs selected by *Sum* conversion types + *t*-test, the others as well.

## Conclusion

In this paper, we propose a fast approach based on the combination of *abs* conversion type and *t*-test, to detect gene–gene synergy. We find that dendrogram-based *I*(*X*_1_; *X*_2_; *Y*) and *doublets* are helpless for discovering pair-wise gene interactions, and the synergic genes selected by our method and the MIC(*X*_1_; *X*_2_; *Y*) method are consistent with the typical pair-wise synergy. However, MIC(*X*_1_; *X*_2_; *Y*) has a higher computational cost. For example, the running time of the entire process on Prostate1 dataset (12,600 × 12,599/2 gene pairs) by MIC(*X*_1_; *X*_2_; *Y*) method is approximately 20 hours (Intel Core i5-4590@3.3 GHz), whereas it is only 47 minutes by our method. Experiments on simulated and real-world data showed that combining the individually discriminant genes selected by *t*-test and the synergic genes selected by our methods can improve prediction performance. These synergic genes should be converted into new variables (*Z*) prior to be used as input features for classifiers.
